# Ostéogenèse imparfaite type III de découverte tardive: à propos d'un cas

**DOI:** 10.11604/pamj.2015.20.14.5382

**Published:** 2015-01-06

**Authors:** Wafae Rachidi, Kawtar Nassar, Saadia Janani, Ouafa Mkinsi

**Affiliations:** 1 Service de Rhumatologie, Centre Hospitalier Universitaire Ibn Rochd, Casablanca, Maroc

**Keywords:** Ostéogenèse imparfaite, fractures, pathologie hétérogène, bisphosphonates, ostéodensitométrie, osteogenesis imperfecta, fractures, heterogeneous condition, bisphosphonates, osteodensitometry

## Abstract

Patient âgé de 52 ans, était admis dans notre établissement pour des polyarthralgies chroniques, plutôt mécaniques, touchant spécialement les articulations du membre inférieur. L'interrogatoire retrouvait l'antécédent de fractures répétées depuis l'enfance, pour des traumatismes minimes, suite à des chutes par glissement. Les signes cliniques et radiologiques, notamment, les fractures récurrentes, sclérotiques bleues, hyperlaxité ligamentaire, dentinogenèse imparfaite, syndrome dysmorphique, ostéoporose densitométrique importante, plaidaient tous en faveur d'une ostéogénèse imparfaite type III. Le patient a initié le traitement, par prise régulière du calcium (1g/jour), supplémentation en vitamine D, et il est programmé pour perfusion de Bisphosphonate (Pamidronate 60mg). Ce cas illustre une forme rare de découverte tardive d'ostéogénèse imparfaite type III.

## Introduction

L'ostéogenèse imparfaite est une maladie héréditaire rare, anciennement appelée maladie de Lobstein ou « maladie des os de verre » [1]. Elle correspond à une maladie unique dont huit formes, avec une présentation clinique et un substrat génétique variables, ont été décrites dans la littérature depuis 1979 [2]. Majoritairement due à une mutation dans l'un des deux gènes qui code pour les chaines alpha du collagène de type I (Col1A1, Col1A2). La fragilité osseuse congénitale qui s'en suit se traduit par des fractures pathologiques, de sévérité très variable: Fractures prénatales et décès périnatal ou formes très frustres [3]. A ces caractéristiques se joignent de façon variable; une petite taille, des sclérotiques bleues, une hyperlaxité cutanée et ligamentaire, une dentinogenèse imparfaite, des déformations des os longs et du rachis, la presence d'os wormiens dans les sutures crâniennes et une surdité à l’âge adulte. Le traitement repose sur une approche multidisciplinaire: les bisphosphonates (ont modifié l évolution de la maladie, surtout chez l'enfant), chirurgie et rééducation physique. Nous rapportons un cas d'ostéogenèse imparfaite de découverte tardive, suite à une hospitalisation pour des arthralgies chroniques.

## Patient et observation

**Figure 1:**
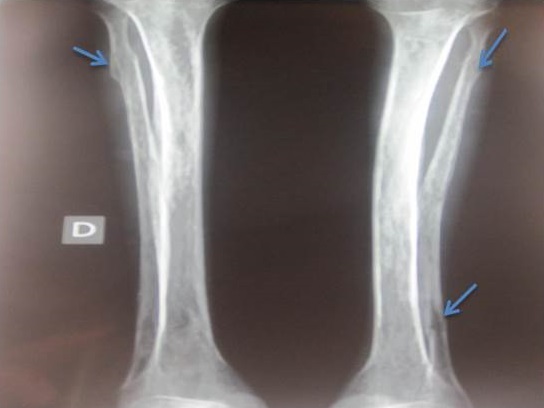
Fracture de l'extrémité supérieure du péroné droit et fracture de l'extrémité supérieure et inférieure du péroné gauche

**Figure 2:**
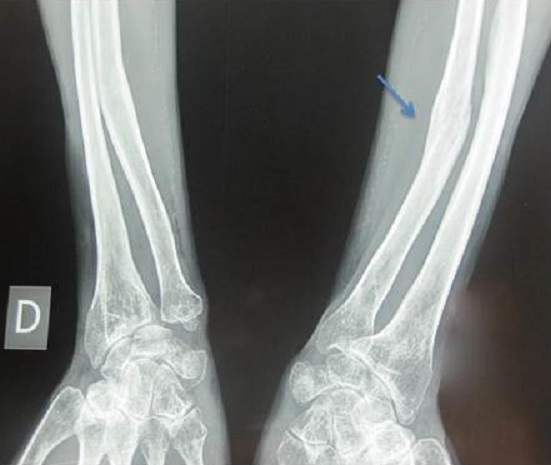
Fracture cubitale gauche

**Figure 3:**
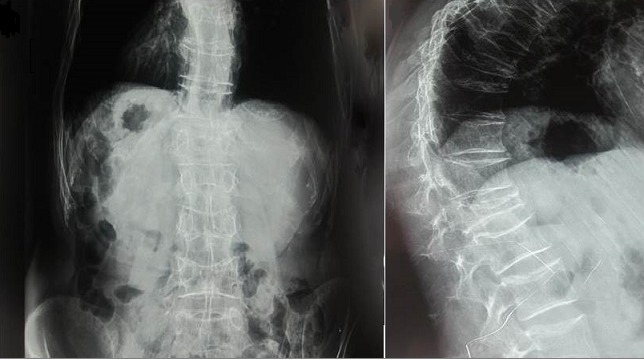
Cyphoscoliose à convexité gauche et fractures vertébrales étagées

Il s'agit d'un patient âgé de 52 ans, sans notion de consanguinité chez les parents, avec développement normal dans sa première enfance. Le patient était admis au service pour des arthralgies mixtes, à prédominance mécanique, débutant aux membres inférieurs. On retrouvait dans ses antécédents; une fracture du coude gauche, à l’âge de 10 ans, suite un glissement, négligée. Deux ans plus tard, des fractures costales étagées, suite une chute de sa hauteur, et à l’âge de 42 ans, une chute de sa hauteur par instabilité occasionnant une fracture de l'extrémité supérieure du péroné droit traitée par plâtre pelvipédieux, mal consolidée. L'examen clinique a mis en évidence: Le front large et légèrement bombé, les sclérotiques bleues, un mauvais état bucco-dentaire. Le thorax avait l'aspect en entonnoir, disproportionné par rapport aux membres longs et grêles, une crosse tibiale bilatérale à convexité antérieure. Les articulations étaient libres et indolores sauf un flessum à 10° irréductible du coude gauche, et des genoux secs, discrètement douloureux à la flexion. On notait également une hyperlaxité ligamentaire. L'examen du rachis retrouvait une cyphoscoliose dorsale à convexité gauche. Les pieds étaient symétriquement plats. Le bilan phosphocalcique notait une calcémie corrigée à 100 mg/l, calciurie à 111 mg/24h, phosphorémie à 28mg/l, phosphaturie à 440 mg/24h, PAL à 353 UI/l, PTH à 46 pg/ml, LDH à 284 UI/l, TSH normale, une hypovitaminose D à 20 ng/ml. L'ensemble du bilan métabolique et inflammatoire était normal. Radiologiquement, il existait des fractures costales et vertébrales étagées sur des os très déminéralisés, une fracture de l'extrémité supérieure du péroné droit et supérieure ainsi qu'inférieure à gauche, une fracture cubitale gauche, une cyphoscoliose dorso-lombaire (Figure 1, Figure 2, Figure 3). L'ostéodensitométrie biphotonique aux rayons X a objectivé une ostéoporose importante avec un T-Score au rachis lombaire à -5.3 (DMO = 0.557 g/cm^2^), au col fémoral T score = - 3.6 (DMO= 0.571), et à l'avant bras T score = -5.2 (DMO = 0.385). La consultation cardiologique était normale et celle oculaire a retrouvé un syndrome sec.

Ainsi, après réunion des arguments cliniques et radiologiques, le diagnostic de l'ostéogenèse imparfaite stade III de Silence a été confirmé chez notre patient. Il fut mit sous calcium (1g/jour), et une supplémentation en vitamine D (100 000UI de cholecalciférol à 15 jours d'intervalle, puis relais par calcifédiol à 400 UI/jour pendant 4 mois), associées à des soins dentaires. Il est programmé pour perfusion de Bisphosphonates (Pamidronate 60 mg/jour, 3 jours de suite).

Par ailleurs, l'examen radiologique des articulations douloureuses a retrouvé des signes d'arthrose grade II de Kallegren Lawrence, ayant nécessité un traitement symptomatique, associé un anti-arthrosique d'action lente oral.

## Discussion

L'ostéogenèse imparfaite (OI) constitue la cause la plus fréquente de l'ostéoporose génétique [4]. C'est une maladie rare, dont la prévalence est de 1 pour 10 000 à 20 000 personnes [5]. Elle touche aussi bien l'homme que la femme, sans prédominance ethnique. Il s'agit d'un désordre autosomique dominant, dû à une mutation du gène du COL 1A1 au niveau du chromosome 17 ou COL 1A2 du chromosome 7 [6]. Cependant, les phénotypes varient considérablement, en fonction des chaînes affectées, la position de la structure du collagène dans laquelle la mutation se produit, et la nature du substituant de l′acide aminé. Certaines formes d′OI de transmission autosomique récessive ont été identifiées. Celles-ci comprennent les défauts de chaperons de collagène et des protéines qui sont impliquées dans l′assemblage duprocollagène de type I, ainsi que dans les protéines qui sont impliquées dans la formation et de l′homéostasie du tissu osseux [7].

L'ostéogenèse imparfaite présente un spectre clinique large, semblable à d′autres affections touchant le tissu conjonctif, telles que le syndrome de Marfan ou le syndrome d'Ehlers-Danlos [8]. Néanmoins, il existe des caractéristiques squelettiques et extra-squelettiques distinguant la maladie,telles qu'une susceptibilité accrue aux fractures dont la sévérité est très variable, des signes cliniques typiques; comportant des sclérotiques bleues, une dentinogenèse imparfaite, une hyperlaxité cutanée et ligamentaire,des troubles de l′audition, une petite taille et des déformations osseuses. Il en résulte également des anomalies de la coagulation et de la cicatrisation, une élévation du métabolisme basal, l′obstruction des voies aériennes ainsi que des anomalies cardiovasculaires. Deux cas d'atteinte rénale, à type d'hypercalciurie ont été publiés. L'atteinte neurologique peut se manifester par des céphalées, une atteinte des derniers nerfs crâniens, une hydrocéphalie avec dilatation ventriculaire et une hyper-réflexie [9]. La plupart des études ont rapporté une variabilité et hétérogénéité clinique de la maladie [10]. Ainsi, Sillence et al l'ont classée en quatre types (classification la plus employée), se présentant comme suit: Type I, sévérité clinique légère; type II, mort périnatale; type III, déformations sévères; et type IV, déformations modérées [2]. Cependant, du fait du nombre croissant des cas d'ostéogenèse imparfaite identifiés, Glorieux a ajouté trois autres groupes de patients en fonction de la gravité clinique et les caractéristiques génétiques, défini par: type V, déformations modérées avec aspect normal des sclérotiques et dentaires; type VI, déformations modérées à sévères avec accumulation d'os ostéoïde et état normal des sclérotiques et dentaires; type VII, similaire au type II avec humerus et femur courts et coda vara [11]. Dans le type VIII, les patients présentent un défaut de croissance et de minéralisation osseuse. Aucun consensus n'existe sur les critères diagnostiques. Ces derniers se basent principalement sur les signes et les symptômes qui sont mentionnés ci-dessus, en particulier la présence de la sclérotique bleue et de la dentinogenèse imparfaite. L'analyse radiologique des canaux pulpaires peut être contributive. L'histoire familiale similaire rend le diagnostic très probable, mais peut être difficile sans signes extra-squelettiques ou quand la fragilité osseuse n'est pas franche, d'où l'intérêt d'une évaluation rigoureuse de la densité minérale [12]. Les fractures multiples sans ostéoporose (syndrome de Silverman, syndrome de l'enfant battu) constituent des causes fréquentes de fractures durant la première année de vie, et les ostéoporoses primitives de l'enfant (idiopathique juvénile, osteoporose secondaire de l'enfant, pseudogliome, syndrome de Col-Carpenter, dysplasie fibreuse panostotique, hyper ou hypo phosphatasie, syndrome de Bruck), sont les principaux diagnostics différentiels [13].

Une fois le diagnostic de l'OI est mis en place, une évaluation du patient par une équipe multidisciplinaire est nécessaire. Le traitement aux multiples facettes, repose sur la physiothérapie, la réadaptation et la chirurgie orthopédique, qui représentent les piliers de la gestion de la maladie. Le but du traitement multimodal est de maximiser la mobilité et les capacités fonctionnelles des patients. Sa prise en charge chez l'adulte n'est pas codifiée. Les bisphosphonates administrés par voie intraveineuse semblent apporter les mêmes bénéfices que chez l'enfant. Ces derniers, oraux et intraveineux, bien qu'ils ne sont pas curatifs, constituent des agents anti-résorptifs puissants, confirmés par études histomorphométriques, et complément efficace à des soins complets, largement administrés pour le traitement de tous les types d′OI. Les essais cliniques ont montré leur efficacité dans l′amélioration de la DMO et la rémission des symptômes cliniques [14]. La majorité des études rapportent ces résultats, sans entraver la croissance, par des cures de pamidronates chez l'enfant, administrées annuellement à 90 mg/Kg. Les indications retenues chez l'enfant sont la présence d'au moins 2 fractures annuelles des os longs et apparition de fractures vertébrales. Dans les formes modérées, les traitements oraux sont à l’étude, malgré que la pertinence de leur utilisation par les patients avec les symptômes plus légers et les effets secondaires restent encore inconnus. Par ailleurs, d'autres thérapeutiques médicales sont disponibles, tels que l'hormone de croissance; toutefois, leur efficacité et les effets indésirables associés à leur utilisation nécessitent une évaluation plus approfondie. Le traitement chirurgical est une alternative après échec des traitements précédents (clous centromédullaires, chirurgie suivie de traction pour déformations vertébrales évoluées) [15]. Le pronostic fonctionnel dépend de la sévérité de l'atteinte et de sa prise en charge. Le pronostic vital est lié à l'atteinte respiratoire corrélée à la sévérité des déformations rachidiennes. L'espérance de vie est normale pour le type I ou IV, et plus sévère pour l'ostéogenèse imparfaite plus avancée. Notre patient regroupe les critères retrouvés dans le type III de Sillence de la maladie. La particularité de notre observation réside d'une part, dans sa rareté, ensuite dans sa forme de découverte tardive. Cela appuie l'importance d'un bilan osseux chez tout enfant accusant des douleurs osseuses même en l'absence de fractures.

## Conclusion

L'ostéogenèse imparfaite est une maladie génétique très rare. Afin de ne pas courir le risque d'isolement social pour les enfants et leurs parents, lié aux problèmes spécifiques à la maladie, une approche diagnostique, éducative, et thérapeutique multidisciplinaire doit encadrer la prise en charge de cette affection. L'approche psychosociale devrait être systématique,ainsi que le dépistage prénatal par échographie en plus d'un conseil génétique pour les descendants de patients atteints. Si les critères diagnostiques et les approches thérapeutiques ont vu le jour, un registre de patients atteints de la maladie est souhaitable pour éviter les retards diagnostiques et les évolutions négligées.
